# Education Research: Validation of an AI-Powered Educational Platform for Neuroanatomical Localization and Stroke Syndrome Recognition: A Multicenter Study

**DOI:** 10.1212/NE9.0000000000200350

**Published:** 2026-08-17

**Authors:** Thales Pardini Fagundes, João Brainer Clares de Andrade, Alice Marilia Silva Abreu Mota, Victor Dyego Mena Romeiro Nishimura, Felipe Hideki Soga, Millene Camilo

**Affiliations:** 1 Department of Neurosciences and Behavioral Sciences, Ribeirão Preto Medical School, University of São Paulo, Brazil;; 2 Department of Health Informatics; Department of Neurology, Universidade Federal de São Paulo, Brazil;; 3 Academic Research Organization, Hospital Israelita Albert Einstein, Sao Paulo, Brazil;; 4 Bioengineering Laboratory, Institute of Aeronautics Technology, Sao Jose dos Campos, Brazil;; 5 Department of Radiology and Diagnostic Imaging, Universidade Federal de São Paulo, Brazil;; 6 Department of Radiology and Diagnostic Imaging, Hospital Beneficência Portuguesa de São Caetano do Sul, Brazil; and; 7 Department of Medicine, Centro Universitário São Camilo, Sao Paulo, Brazil.

## Abstract

**Background and Objectives:**

Neuroanatomical localization is foundational to stroke practice but remains difficult across training levels. We evaluated whether LouiS, an AI educational platform using retrieval-augmented generation over a curated stroke-syndrome knowledge base, improves the proportion of correct responses for neuroanatomical localization and stroke-syndrome recognition, with a focus on its potential as a teaching aid.

**Methods:**

This two-phase study recruited 80 participants from 3 Brazilian university-affiliated centers. Phase 1 used web-app allocation with browser-fingerprint persistence to keep each participant in the same test condition, with or without LouiS, across repeated access. Phase 2 used a within-participant paired design in which 62 participants evaluated case-matched vignettes first without and then with LouiS. Participant-level scores used the actual number of scorable responses in each condition. The allocation protocol required exposure to higher-difficulty vignettes and supplemented them with randomly selected cases. The primary outcome was the proportion of correct responses against a consensus reference standard.

**Results:**

Across the pooled response-wise dataset including phase 1 and available phase 2 responses, correct responses increased from 63.4% without LouiS to 71.9% with LouiS (absolute difference 8.5 percentage points; 95% CI 2.79–14.20; generalized estimating equation OR 1.36, 95% CI 1.11–1.67; *p* = 0.003). In phase 2, median participant-level proportion correct increased from 62.1% [IQR 60.0–80.0] without LouiS to 80.0% [IQR 60.0–100.0] with LouiS, a mean within-participant gain of 7.9 percentage points (95% CI 4.10–11.75; Wilcoxon W = 23.0, *p* = 0.0002; Cohen *d* = 0.53). In paired response-wise analysis, 30 responses changed to correct and 5 to incorrect (McNemar χ^2^ = 16.46, *p* < 0.0001). Deviations clustered on differential-diagnostic pairs.

**Discussion:**

Use of LouiS was associated with improved performance in neuroanatomical localization and stroke-syndrome recognition during case-based testing. The findings support LouiS as an educational aid for neuroanatomical reasoning and continuing medical education. Knowledge retention, behavior change, and real-world clinical outcomes require separate prospective evaluation.

## Introduction

Neuroanatomical localization, the systematic inference of lesion location from clinical signs and symptoms, remains a cornerstone of neurologic practice, yet mastering it requires extensive training and repeated exposure to varied cases.^1,2^ In acute stroke care, accurate topographic diagnosis directly influences treatment selection and interpretation of imaging findings.^3^ Despite its importance, clinicians across expertise levels face persistent difficulty synthesizing complex presentations into coherent neuroanatomical frameworks, particularly for uncommon posterior-circulation, brainstem, and alternating syndromes.^1,2^

Artificial intelligence in stroke and emergency medicine has been applied mainly to automated neuroimaging interpretation, particularly detection of intracranial hemorrhage and large-vessel occlusion.^4-9^ In contrast, few AI tools are designed to support bedside neuroanatomical reasoning from clinical presentation rather than from imaging alone.^10,11^ Neuroanatomical reasoning is also a teaching problem: learners must connect clinical signs, vascular territories, and lesion sites before they can interpret imaging with confidence.^12,13^

Retrieval-augmented generation (RAG) combines a language model with retrieval from a curated knowledge base.^14-17^ For neuroanatomical localization, the main value of RAG is not rapid updating of unstable therapeutic knowledge. The relevant value is coverage of rare syndromes, transparent retrieval of the syndrome entries used to generate an answer, and standardized explanatory language that can be used in case discussion.^15-19^ This is relevant because general-purpose large language models can encode clinical knowledge but remain vulnerable to unsupported generation and hallucination.^20-24^

We developed LouiS (Localization and Stroke Syndromes System), a web-based AI educational platform for neuroanatomical localization and stroke-syndrome recognition. The platform is freely available online.^25^ LouiS retrieves structured syndrome entries from a 619-entry multilingual knowledge base and uses Gemini Flash 3 to generate ranked diagnostic candidates with anatomical and vascular localization. The intended use of the platform in this manuscript is educational and case based; it is not presented as an autonomous clinical decision maker.

We aimed to evaluate whether use of LouiS improves the proportion of correct responses for neuroanatomical localization and stroke-syndrome recognition compared with unaided case assessment across clinicians with different levels of expertise.

## Methods

### Study Design and Setting

This was a two-phase, prospective study evaluating the association between use of LouiS and the proportion of correct responses for neuroanatomical localization and stroke-syndrome recognition. Clinical vignettes were presented in Brazilian Portuguese, reflecting routine clinical language and training environments. Representative vignettes and supplementary analyses are provided in the Supplement; the platform version history is provided in eAppendix 1.

### Participants and Recruitment

This was a convenience sample. Participants were recruited from 3 university-affiliated centers in Brazil through institutional channels, messaging applications, and direct invitation by the investigators. Eligible participants included medical students from the fourth year onward, neurology or neurosurgery residents, general practitioners, general neurologists, and vascular neurologists. Participation was voluntary, digital informed consent was obtained before response submission, and no participant was required to complete both phases.

### Case Development and Reference Standard

All clinical cases and corresponding answer keys were defined a priori by a panel of 3 board-certified vascular neurologists who were authors of the project (T.P.F., J.B.C.A., and M.R.C.). Each panelist independently reviewed the clinical vignettes and proposed diagnoses, neuroanatomical localizations, and syndromic classifications. Discrepancies were resolved through structured discussion, and the final reference standard was established by full consensus. The same consensus answer key was applied in both phases.

### LouiS Platform Development

LouiS was developed as an educational platform grounded in published evidence on stroke syndromes and neurologic localization. The knowledge base was derived from peer-reviewed literature published between 1990 and 2025. Overall, the platform includes 619 structured entries with syndrome names, aliases, symptoms, vascular territories, and anatomical localizations in Portuguese, English, and Spanish.

### Platform Architecture

During inference, the clinical text is tokenized after language-specific stop-word filtering. Tokens are matched against an inverted keyword index covering syndrome names, aliases, symptoms, anatomical locations, and vascular territories. The top-ranked retrieved syndrome entries are passed as structured context to Gemini Flash 3 with a constrained JSON output schema, which returns ranked candidate syndromes, anatomical localization, vascular territory, and explanatory text.

This architecture prioritizes interpretability and traceability: each ranked suggestion is linked to structured entries retrieved from the curated knowledge base, and the model's role is to format and explain the retrieved content for educational use.

Retrieved entries, therefore, serve as an inspectable teaching substrate for the language model. The trainee can compare the clinical signs in the vignette with the retrieved syndrome descriptions and see why a syndrome was suggested.

The platform separates retrieval from language generation: the syndrome candidates come from the curated knowledge base, while the language model formats and explains the output. This design reduces reliance on unsupported free-form generation and allows targeted updates to the knowledge base.

### Neuroimaging-Based Anatomical and Vascular Mapping

To support precise neuroanatomical localization, 2 board-certified neuroradiologists independently reviewed normal brain MRI studies, including axial, coronal, and sagittal sequences, to define 40 anatomically and clinically relevant sites spanning the cerebral hemispheres, cerebellum, and brainstem. These sites were selected to represent canonical regions involved in common vascular and topographic neurologic syndromes.

Selected anatomical regions were manually annotated using translucent hatching overlays, allowing visualization of region boundaries while preserving underlying anatomical detail. An analogous process was applied to normal intracranial MR angiography studies, in which the major intracranial arteries were identified and annotated using the same translucent overlay technique.

Final selection of anatomical sites and vascular structures was determined by consensus between the 2 neuroradiologists, following joint review and resolution of discrepancies. These annotated neuroimaging templates were incorporated into the LouiS platform as visual and conceptual anchors for anatomical and vascular reasoning.

### Integration Into the Decision-Support Workflow

The literature-derived syndrome index, retrieved structured context, and neuroimaging-derived anatomical and vascular maps were integrated into LouiS to support case-based educational feedback for neuroanatomical localization and stroke-syndrome recognition. Users retained full autonomy to accept, modify, or reject platform outputs.^26-28^

### Study Procedures

#### Phase 1: Browser-Fingerprint Allocated Assessment

At first access, participants were assigned by the web application to evaluate cases either with or without LouiS. A browser-fingerprint identifier preserved the same assigned condition across repeated access and reduced accidental rerandomization. Because responses were not necessarily paired within participants, phase 1 data were analyzed primarily as response-wise data.

#### Phase 2: Within-Participant Paired Assessment

In phase 2, a subset of phase 1 participants (n = 62) completed an additional paired evaluation. Each participant evaluated case-matched vignettes first without LouiS and then with LouiS, with theion to disagree with the platform output. The allocation protocol required exposure to higher-difficulty vignettes and supplemented them with randomly selected cases; the case set was case-matched within each participant but was not necessarily identical across all participants. Participant-wise performance was computed as the proportion of correct responses among the scorable cases each participant completed in a given condition; most participants completed 5 cases per condition, but the denominator varied in a small subset because some participants had partial or additional scorable responses.

### Outcomes

The prespecified primary outcome was the proportion of correct responses. At the response level, the outcome was coded as correct or incorrect against the consensus reference standard. At the participant level (phase 2), performance was the proportion of correctly classified cases per participant in each condition. This proportion used the actual number of scorable responses in that condition as the denominator; therefore, participant-level percentages were not restricted to 20-percentage-point increments. Secondary descriptive outcomes were the postuse questionnaire items: ease of use, perceived speed, and number of answers changed after consulting LouiS. The System Usability Scale was attempted but completed by only 5 participants and was not used for inference.

### Statistical Analysis

The primary analysis was phase 2 participant-wise. Because the Shapiro-Wilk test rejected normality of paired differences (W = 0.756, *p* < 0.001), within-participant comparison used the Wilcoxon signed-rank test. Results are reported with effect sizes and 95% CI. Response-wise comparisons used χ^2^ tests for unpaired comparisons and the McNemar test for paired binary responses. A generalized estimating equation (GEE) model with binomial distribution and exchangeable working correlation accounted for within-participant clustering. The sample size was determined by the number of participants available during the recruitment period. Minimum detectable effects (alpha = 0.05, 80% power) are reported in eTable 3 only to clarify why subgroup findings should be treated as exploratory rather than confirmatory.

All tests were two-sided. Results are reported as medians with interquartile ranges, mean differences with 95% CI, odds ratios (OR) with 95% CI, and exact *p* values where appropriate. Complete-case analysis was used without imputation. Statistical analyses were performed using SPSS version 29.0 and Python 3.12 in Google Colaboratory.

### Standard Protocol Approvals, Registrations, and Patient Consents

The study protocol was reviewed and approved by the Research Ethics Committee of Hospital das Clínicas da Faculdade de Medicina de Ribeirão Preto, University of São Paulo (HCFMRP-USP; CAAE 86496224.0.1001.5440). The study protocol was reviewed. All procedures were conducted in accordance with institutional and national ethical standards. Digital informed consent was obtained from all participants before response submission.

### Data Availability, Code, and Supplementary Materials

Deidentified participant data will be available from the corresponding author upon reasonable request after publication for approved noncommercial academic analyses, subject to institutional ethics and data-protection review. The structured stroke-syndrome knowledge base is available in Mendeley Data (doi: 10.17632/r5k3nzb8gx.1). The public LouiS interface is available online.36 Examples of clinical vignettes, supplementary analyses, the version-history appendix, and the corrected supplementary tables are provided in the Supplement.

## Results

Eighty participants completed phase 1. Among these, 62 completed phase 2 and contributed paired data, whereas 18 contributed only to the phase 1 response-level analyses. Across both phases, the aggregated response-level dataset comprised 1,024 individual case responses (536 without LouiS and 488 with LouiS).

**Figure 1:**
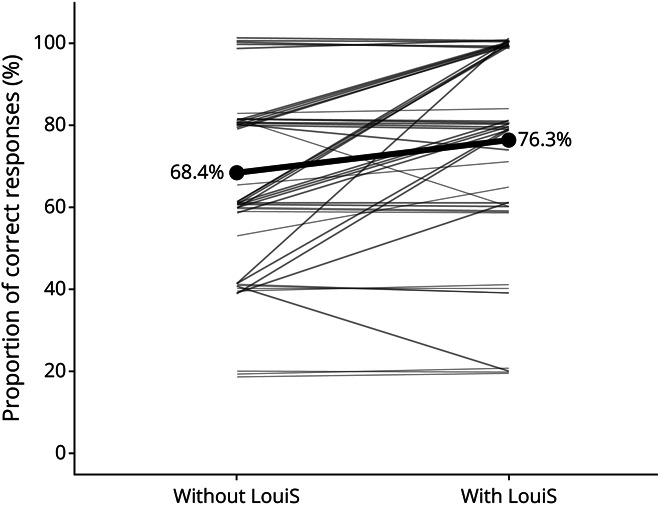
Each Line Represents 1 Participant Who Completed Phase 2 Proportion of correct responses, defined as the proportion of correctly classified cases, is shown for evaluations performed without LouiS (left) and with LouiS (right). Lines illustrate within-participant changes. Black dots show mean participant-level proportion correct, which increased from 68.4% without LouiS to 76.3% with LouiS. Participant-wise medians are reported in Table 1. The figure is interpretable in grayscale; color is not required.

In phase 2 (paired analysis), median participant-level proportion of correct responses increased from 62.1% [IQR 60.0–80.0] without LouiS to 80.0% [IQR 60.0–100.0] with LouiS, corresponding to a mean within-participant improvement of 7.9 percentage points (95% CI 4.10–11.75; Wilcoxon W = 23.0, *p* = 0.0002; median paired difference 0.0 [IQR 0.0–20.0]; Cohen *d* = 0.53; Table 1). Thirty-eight participants (61.3%) showed no change, 21 (33.9%) improved, and 3 (4.8%) worsened. Individual paired changes are shown in Figure 1; the figure labels the mean participant accuracy (68.4% without LouiS and 76.3% with LouiS), whereas Table 1 reports the medians.

**Table 1:** Participant-Level Proportion of Correct Responses in Phase 2 Paired Analysis

Population/professional group	n	Proportion of correct responses without LouiS, median [IQR], %	Proportion of correct responses with LouiS, median [IQR], %	Mean paired difference, percentage points	95% confidence interval	Wilcoxon signed-rank test (*p*)
All participants	62	62.1 [60.0 to 80.0]	80.0 [60.0 to 100.0]	7.9	4.10 to 11.75	0.0002
Medical students	15	60.0 [57.2 to 80.0]	63.6 [60.0 to 80.0]	4.6	0.01 to 9.20	0.059
Neurology and neurosurgery residents	14	80.0 [60.0 to 80.0]	80.0 [63.8 to 95.0]	5.4	−4.28 to 15.00	0.276
Neurologists, general	6	80.0 [65.0 to 95.0]	90.0 [65.0 to 100.0]	3.3	−5.02 to 11.68	1.000
Neurologist experts, vascular	18	72.2 [60.0 to 95.0]	80.0 [73.6 to 100.0]	7.1	1.10 to 13.00	0.024
General practitioners	9	60.0 [40.0 to 60.0]	100.0 [80.0 to 100.0]	22.2	4.40 to 40.00	0.047

Population	n	Without LouiS, mean (SD), %	With LouiS, mean (SD), %	Mean paired difference, percentage points	95% confidence interval	Wilcoxon signed-rank test (*p*)
Nonstudent participants	47	69.9 (21.2)	78.9 (22.6)	9.0	4.10 to 13.90	0.0006

Participant-wise proportion of correct responses in phase 2, in which the same individuals evaluated identical clinical cases without and then with LouiS. Performance represents the proportion of correctly classified cases per participant. Participant-level proportions used the actual number of scorable responses each participant completed in a given condition; most participants completed 5 cases per condition, with a small subset having fewer or additional scorable responses, so participant-level proportions are not restricted to 20-percentage-point increments. Values are reported as medians [IQR] or means with 95% confidence intervals as appropriate. Wilcoxon signed-rank *p* values are reported; subgroup analyses are exploratory.

Participant-wise paired effects stratified by professional group are presented in Table 1 as exploratory analyses. The point estimates were +4.6 percentage points for medical students (95% CI 0.01–9.20; *p* = 0.059), +5.4 for neurology and neurosurgery residents (95% CI −4.28 to 15.00; *p* = 0.276), +7.1 for vascular neurologists (95% CI 1.1–13.0; *p* = 0.024), +22.2 for general practitioners (95% CI 4.4 to 40.00; *p* = 0.047), and +3.3 for general neurologists (95% CI −5.02 to 11.68; *p* = 1.000). Only the vascular-neurologist and general-practitioner comparisons reached statistical significance; the medical-student, resident, and general-neurologist comparisons did not. Because the study was not powered for subgroup inference, these results are used only to describe possible patterns for future educational studies.

As a sensitivity analysis, excluding medical students (n = 47) yielded a mean paired improvement of 9.0 percentage points (95% CI 4.10–13.90; *p* = 0.0006), consistent with the primary analysis.

In the pooled response-wise analysis, the proportion of correct responses increased from 63.4% without LouiS to 71.9% with LouiS, corresponding to an absolute difference of 8.5 percentage points (95% CI 2.79–14.20; χ^2^ *p* = 0.0036; Table 2). A GEE model accounting for within-participant clustering showed an overall response-wise association (OR 1.36, 95% CI 1.11–1.67; *p* = 0.003).

**Table 2:** Response-Level Proportion of Correct Responses in Phase 1 and Combined Analyses, Overall and by Professional Group

Population/professional group	Responses without LouiS	Responses with LouiS	Proportion of correct responses without LouiS, %	Proportion of correct responses with LouiS, %	Mean difference, percentage points	95% confidence interval	Chi-square test (*p*)
All responses	536	488	63.4	71.9	8.5	2.79 to 14.20	0.0036
Medical students	81	81	62.7	64.2	1.5	−13.29 to 16.38	0.838
Neurology and neurosurgery residents	149	171	68.5	69.0	0.5	−9.67 to 10.77	0.916
Neurologists, general	82	60	50.0	75.0	25.0	14.58 to 35.42	0.0026
Neurologist experts, vascular	177	135	67.8	77.8	10.0	0.12 to 19.88	0.047
General practitioners	45	41	55.6	75.6	20.1	0.24 to 39.87	0.051

Abbreviation: GEE = generalized estimating equation.

Response-wise proportion of correct responses comparing case responses provided with and without LouiS. Responses include phase 1 and phase 2 data; therefore, pooled response-wise analyses are secondary. Differences represent absolute percentage-point changes with 95% confidence intervals. GEE results account for within-participant clustering. Paired phase 2 binary changes are reported separately using McNemar testing.

When restricted to phase 2 participants only, the response-wise difference was 5.7 percentage points and did not reach statistical significance (*p* = 0.098); the pooled response-wise result is, therefore, secondary and partly reflects inclusion of phase 1–only responses. In the paired binary subset, 30 responses changed from incorrect to correct and 5 from correct to incorrect (McNemar χ^2^ = 16.46, *p* < 0.0001).

Among 52 participants who completed the postuse questionnaire, 32 (61.5%) rated the platform as easy to use, 18 (34.6%) as partially easy, and 2 (3.8%) as not easy. Eighteen participants (34.6%) reported being faster with LouiS, 23 (44.2%) reported no difference, and 11 (21.2%) found it slower. Participants changed a mean of 1.4 responses after consulting LouiS.

The pattern-of-deviations analysis evaluated where final participant answers differed from the consensus reference answer. In phase 2, 181 deviations were observed. They clustered on differential-diagnostic pairs, including Benedikt vs Claude syndrome, Heubner vs anterior cerebral artery infarction, lateral superior cerebellar artery (SCA) vs SCA/Anterior Inferior Cerebellar Artery (AICA) overlap, Wallenberg vs hemimedullary syndrome, and alexia without agraphia vs visual agnosia. These deviations reflect participant final answers with access to LouiS V3, not autonomous platform accuracy.

Baseline performance was inversely associated with gain after LouiS exposure (Pearson *r* = −0.26, *p* = 0.043; Spearman rho = −0.27, *p* = 0.033), suggesting that participants with lower baseline accuracy were more likely to benefit.

**Figure 2:**
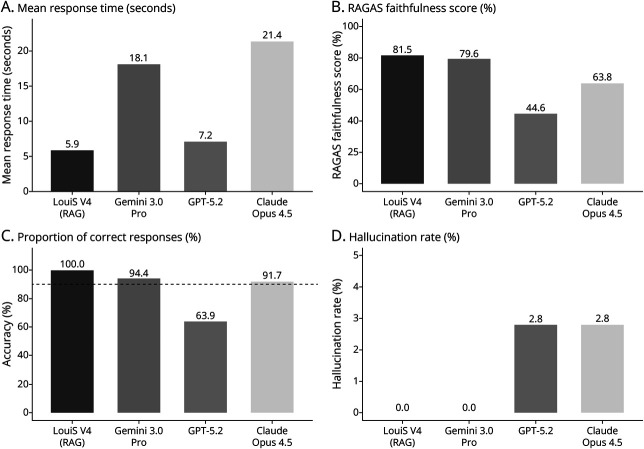
Four-Panel Bar Chart Comparing LouiS V4 Against Gemini 3.0 Pro, GPT-5.2, and Claude Opus 4.5 Across 36 Validation Cases Panels show (A) mean response time (s), (B) RAGAS faithfulness score (%), (C) accuracy (%), and (D) hallucination rate (%). LouiS V3 was used in the participant validation, whereas LouiS V4 was used for this automated benchmark. The benchmark was separate from participant testing and should not be interpreted as Phase 2 participant performance or Phase 2 platform-output accuracy; mean participant-level proportion correct with LouiS during Phase 2 was 76.3%. The figure is interpretable in grayscale; color is not required.

In a separate automated benchmark using 36 clinical vignettes, LouiS V4 achieved 100% standalone accuracy as a system-level measure based on direct platform queries and top-ranked V4 outputs, compared with 94.4% for Gemini 3.0 Pro, 91.7% for Claude Opus 4.5%, and 63.9% for GPT-5.2. The hallucination rate was 0% for LouiS and Gemini 3.0 Pro and 2.8% for GPT-5.2 and Claude Opus 4.5. Figure 2 illustrates the benchmark. Phase 1 and phase 2 used LouiS V3, whereas the automated benchmark used LouiS V4. This benchmark accuracy should not be interpreted as phase 2 participant performance or phase 2 platform-output accuracy; mean participant-level proportion correct with LouiS during phase 2 was 76.3%.

## Discussion

In this multicenter two-phase study, the use of LouiS was associated with improved performance in neuroanatomical localization and stroke-syndrome recognition during paired case testing. This result may be interpreted within an educational context of a case-based platform for neuroanatomical reasoning, rather than as evidence supporting autonomous clinical decision making.

The pattern of improvement suggests where the platform may be most useful educationally. General practitioners had the largest gain, consistent with clinicians who have general clinical reasoning skills but less repeated exposure to vascular neuroanatomical syndromes. Vascular neurologists also improved modestly, probably because the test included uncommon syndromes and posterior-circulation localizations. General neurologists had unstable estimates because of the very small subgroup size. Students improved less, which may indicate that the platform is easier to use after a minimum level of clinical examination and localization knowledge has been acquired.

The deviation analysis provides the clearest educational signal in the dataset. If LouiS and the expert reference standard were correct, then deviations represent clinically meaningful reasoning errors or deliberate disagreements. These deviations clustered on classical diagnostic boundaries rather than on random labels. Such patterns are useful for teaching because they identify where learners confuse adjacent syndromes, vascular territories, or lesion levels.

The study did not test knowledge acquisition, retention, or behavior change. Therefore, the educational claim is deliberately narrow: LouiS improved performance during case-based assessment and made specific localization errors visible. Whether repeated use produces durable learning requires a delayed post-test or longitudinal curriculum study.

Subgroup analyses should be interpreted cautiously because several groups were underpowered. The apparent superiority or inferiority of small groups, especially general neurologists, should not be overinterpreted.

For neuroanatomical reasoning, RAG is useful because retrieval makes the reasoning path inspectable and because rare syndromes can be represented explicitly in the knowledge base.^14-17,22-24^ The platform retrieves explicit syndrome entries and then uses language generation to format and explain the answer.

The implemented LouiS architecture uses an inverted keyword index over structured syndrome names, symptom fields, anatomical localizations, and vascular territories. Retrieved entries are passed as context to Gemini Flash 3, which formats the ranked candidates and explanatory text. This separation makes the retrieved source entries inspectable for teaching.

Versioning matters for interpretation. The participant validation used LouiS V3, whereas the automated benchmark used LouiS V4 in a separate postvalidation run. The V4 benchmark is reported as a standalone system-level measure and is not used to infer phase 2 participant or platform-output accuracy.

Moving from vignette-based validation to routine educational deployment will require workflow-fit testing in case-discussion settings, attention to learner acceptance, careful supervision to avoid automation bias, and equity safeguards so that access to digital teaching tools does not widen training disparities.^26-36^

This study has limitations. First, text vignettes do not capture the full complexity of real encounters with incomplete data and evolving findings. Second, all vignettes were in Brazilian Portuguese, limiting generalizability. Third, the reference standard was expert consensus, not imaging-pathologic confirmation. Fourth, phase 2 used a fixed order without counterbalancing. Fifth, the participant validation measured final participant answers with access to LouiS V3 rather than autonomous platform accuracy; individual V3 suggestions could differ from the consensus reference standard, as illustrated by a high-deviation case in eTable 5. Sixth, participants were recruited from university-affiliated centers in Brazil. Seventh, several subgroups were underpowered. Eighth, the literature-derived knowledge base may overweight rare or atypical cases because such cases are more likely to be published. Finally, knowledge retention, behavior change, and clinical outcomes were not measured.

An AI-powered educational platform using retrieval-augmented generation over a curated stroke-syndrome knowledge base was associated with improved performance in neuroanatomical localization and stroke-syndrome recognition during case-based testing. The strongest current use case is teaching: LouiS may help expose learners and clinicians to less familiar localization patterns, make differential-diagnostic errors visible, and structure discussion of difficult vascular syndromes. Knowledge retention and clinical-care effects require separate study. The platform is freely available online.

## Glossary

GEE: generalized estimating equation
OR: odds ratios
RAG: retrieval-augmented generation
SCA: superior cerebellar artery
